# HIV and Aging Comes into the Spotlight: 2012 to 2015

**DOI:** 10.1093/geroni/igaa057.3113

**Published:** 2020-12-16

**Authors:** Mark Brennan-Ing

**Affiliations:** Hunter College, CUNY, New York, New York, United States

## Abstract

I had the privilege of serving as Principal- and Co-Convener of the HIV, AIDS and Older Adults Special Interest Group (SIG) for four years (2012 through 2015). During this era, when the proportion of U.S. older adults with HIV was projected to surpass 50%, we witnessed a number of milestones including the NIH Office of AIDS Working Group on HIV and Aging recommendations for critical research focus publication (JAIDS, 2012), the first CDC Surveillance Report on people 50 and older with HIV (2013), and the first UNAIDS report on HIV and aging (2013). During this period, the SIG was very successful in raising awareness about HIV and aging through numerous GSA presentations. Topics covered ranged from sexual health, to cognitive function, psychological well-being, social isolation, successful aging, and resilience. These presentations highlighted research findings that have been critical in developing interventions and shaping policy initiatives to support this growing population. Part of a symposium sponsored by the HIV, AIDS and Older Adults Interest Group.

